# Intelligence and socioeconomic position in childhood in relation to frailty and cumulative allostatic load in later life: the Lothian Birth Cohort 1936

**DOI:** 10.1136/jech-2015-205789

**Published:** 2015-12-23

**Authors:** Catharine R Gale, Tom Booth, John M Starr, Ian J Deary

**Affiliations:** 1 Department of Psychology, Centre for Cognitive Ageing & Cognitive Epidemiology, University of Edinburgh, Edinburgh, UK; 2 MRC Lifecourse Epidemiology Unit, University of Southampton, Southampton, UK; 3 Geriatric Medicine Unit, University of Edinburgh, Western General Hospital, Edinburgh, UK

**Keywords:** COGNITION, ELDERLY, SOCIAL CLASS, Cohort studies

## Abstract

**Background:**

Information on childhood determinants of frailty or allostatic load in later life is sparse. We investigated whether lower intelligence and greater socioeconomic disadvantage in childhood increased the risk of frailty and higher allostatic load, and explored the mediating roles of adult socioeconomic position, educational attainment and health behaviours.

**Methods:**

Participants were 876 members of the Lothian Birth Cohort 1936 whose intelligence was assessed at age 11. At age 70, frailty was assessed using the Fried criteria. Measurements were made of fibrinogen, triglyceride, total and high-density lipoprotein cholesterol, albumin, glycated haemoglobin, C reactive protein, body mass index and blood pressure, from which an allostatic load score was calculated.

**Results:**

In sex-adjusted analyses, lower intelligence and lower social class in childhood were associated with an increased risk of frailty: relative risks (95% CIs) were 1.57 (1.21 to 2.03) for a SD decrease in intelligence and 1.48 (1.12 to 1.96) for a category decrease in social class. In the fully adjusted model, both associations ceased to be significant: relative risks were 1.13 (0.83 to 1.54) and 1.19 (0.86 to 1.61), respectively. Educational attainment had a significant mediating effect. Lower childhood intelligence in childhood, but not social class, was associated with higher allostatic load. The sex-adjusted coefficient for allostatic load for a SD decrease in intelligence was 0.10 (0.07 to 0.14). In the fully adjusted model, this association was attenuated but remained significant (0.05 (0.01 to 0.09)).

**Conclusions:**

Further research will need to investigate the mechanisms whereby lower childhood intelligence is linked to higher allostatic load in later life.

## Introduction

Frailty is a syndrome of decreased reserve and increased vulnerability to stressors due to age-related impairments in multiple, inter-related systems and a decline in the ability to maintain homeostasis which increases the risk of adverse outcomes.^1^ ^2^ The causes of frailty are likely to involve biological and psychosocial mechanisms.^3^ If frailty is a consequence of cumulative decline in multiple physiological systems,^2^ clues to its aetiology might come from studying determinants of frailty decades before its onset.

Evidence on childhood determinants of frailty is sparse. In Latin America, greater socioeconomic deprivation in childhood was associated with an increased likelihood of frailty.^4^ In France, poorer living standards in childhood were linked with greater frailty, but not independently of markers of socioeconomic position later in life.^5^ Whether childhood socioeconomic inequalities are predictive of frailty in other populations is unknown.

Another childhood factor that might influence frailty risk is intelligence. Higher childhood intelligence has been frequently associated with reduced mortality and morbidity in adult life.^6^ ^7^ These associations do not appear to be confounded by parental socioeconomic position,^6^ ^7^ though childhood intelligence might be acting as a proxy for other aspects of the early social environment not well captured by social class. Various, non-exclusive, mechanisms may underlie these associations, including disease prevention, healthier behaviour, adult socioeconomic advantage, reduced risk of mental disorder and ‘system integrity’—that is, a better functioning brain might indicate a body whose systems act more efficiently.^8^ ^9^ Some of these same mechanisms could link intelligence in childhood with risk of frailty in later life: several risk factors for frailty—such as smoking,^10^ obesity,^11^ adult socioeconomic deprivation,^12^ lower educational attainment^13^—have been associated with lower childhood intelligence.^14–17^ Another possible pathway might be via stress regulation. Cognitive function is thought to influence stress perception,^18^ and the efficacy with which individuals cope with stressors, whether in childhood^19^ or later life.^20^ This might affect health via neuroendocrine or other physiological mechanisms.^21^

Allostasis refers to the temporary adjustments that take place in physiological systems to maintain stability in the face of fluctuation in environmental demands.^22^ Such adaptations are protective in the short term, but in the long term, repeated episodes of allostasis together with inefficient activation or turning off of physiological responses means that the body experiences ‘wear and tear’ of all major regulatory systems which increases the risk of morbidity or death.^23^ ^24^ Allostatic load has been conceptualised as a measure of this cumulative biological dysregulation or wear and tear across regulatory systems.^23^ ^24^ The concept of allostatic load differs from that of frailty, in that it has been applied in studies of people from childhood upwards, rather than just in older populations, but it shares considerable theoretical overlap with frailty, in that cumulative decline in multiple physiological systems is thought to underlie the clinical presentation of this syndrome.^2^

There have been few investigations of the links between allostatic load and frailty, but one longitudinal study found that higher allostatic load was associated with an increased risk of incident frailty,^25^ suggesting that dysregulation in multiple physiological systems may provide a biological warning of frailty risk.^25^

Childhood circumstances or characteristics may have a long-lasting influence on the regulation of physiological systems. In children, allostatic load increases with greater exposure to socioeconomic disadvantage.^26^ The impact on allostatic load of such exposure may still be apparent in adulthood.^27^ Prolonged exposure to socioeconomic disadvantage later in life has also been linked with higher allostatic load.^27^ Lower intelligence in childhood has been linked with contributors to higher allostatic load in adult life, such as higher blood pressure,^28^ body mass index (BMI),^16^ and concentrations of triglycerides,^29^ glucose,^29^ inflammatory and haemostatic factors.^30^ A recent study reported an association between lower childhood intelligence and higher allostatic load at age 73.^31^ Childhood intelligence may be linked to later life allostatic load via the same potential mechanisms specified above in the case of frailty.

We investigated the hypotheses that older people who as children had lower intelligence or were exposed to greater socioeconomic disadvantage would be at greater risk of frailty and have higher allostatic load. We regarded adult social class, educational attainment and health behaviours as potential mediators of any association. Lower intelligence and socioeconomic disadvantage in childhood are consistently associated with lower educational attainment and socioeconomic disadvantage in adult life. Such an environment in adulthood increases the risk of frailty^13^ and, perhaps partly due to its inherent stresses, allostatic load.^27^ ^32^ Lower intelligence and socioeconomic disadvantage in childhood have been linked with poorer health behaviours in adult life.^14^ Potential mechanisms underlying these links may include lower educational attainment and poorer self-management of health risk.^33^ Health behaviours may contribute to allostatic load by altering its biomarkers, and there is some evidence that smoking and lack of physical activity increase the risk of frailty.^10^ ^34^

## Methods

### Participants

The Lothian Birth Cohort 1936 (LBC1936) was set up to study cognitive ageing in surviving members of the Scottish Mental Survey of 1947.^35^ In total 1091 community-dwelling people were recruited at a mean age of 70 years. Ethical approval was obtained from the Multi-Centre Ethics Committee for Scotland and Lothian Research Ethics Committee. The study conformed to the principles embodied in the Declaration of Helsinki.

### Measures

#### Intelligence in childhood

Most children born in 1936 and attending school on 4 June 1947 took the Moray House Test No 12, a test of general intelligence, when they were aged about 11 as part of the Scottish Mental Survey. It was concurrently validated against the Terman-Merrill Revision of the Binet Scales.

#### Social class in childhood and adulthood

Participants provided information on their father's occupation when they were aged 11 years. Occupations were classified into five social class categories: professional, managerial, skilled non-manual, skilled manual, and semiskilled/unskilled. Own socioeconomic position was derived from participants’ (or their spouses’) highest reported occupation and classified into categories as described above.

#### Environmental deprivation in childhood

Participants provided information on living conditions at age 11: the number of people living in their home, the number of rooms, the number of people who shared toilet facilities, and whether these were indoors or outdoors. We calculated number of persons per room, and separately standardised this and the two variables on toilet facilities. We formed a composite measure of environmental deprivation in childhood by summing these standardised variables.

#### Health behaviours

Participants provided information on alcohol intake in the past week, smoking history and physical activity. Physical activity was assessed on a six-point scale ranging from movement associated with necessary (household) chores to keep-fit/heavy exercise or competitive sport. We categorised alcohol intake in three groups: abstainers (no alcohol), or drinkers within or above sex-specific recommended weekly limits (≤21 vs 22+ units for men; ≤14 vs 15+ units for women).^36^

#### Educational attainment

Participants provided information on highest educational qualification. This was categorised as: no qualifications, O level or equivalent, A level or equivalent, semiprofesssional or professional qualifications, degree.

#### Frailty

Maximum handgrip strength was measured three times on each side using a dynamometer; the best of these measurements was used for analysis. BMI was calculated as weight (in kilograms)/height (in metres)^2^. Gait speed was assessed by measuring time taken to walk 6 m at maximum speed. Participants were asked to indicate their usual level of physical activity on a six-point scale, ranging from ‘moving only in connection with necessary (household) chores’ to ‘keep-fit/heavy exercise or competitive sport several times a week’. Symptoms of depression were assessed using the depression subscale of the Hospital Anxiety and Scale (HADS-D).^37^ We used these data to derive indicators of frailty or prefrailty using the Fried criteria.^1^ Physical frailty is defined as the presence of three or more of: unintentional weight loss, weakness, self-reported exhaustion, slow walking speed and low physical activity. Prefrailty is defined as the presence of one or two of these criteria. We operationalised these criteria using definitions similar to those used in Fried's original studies: weight loss was defined as current BMI <18.5 kg/m^2^; weakness was defined as maximum grip strength in the lowest 20% of the distribution, taking account of sex and BMI; exhaustion was considered present if the participant responded positively to the HADS-D question ‘I feel as if I'm slowed down’; slow walking speed was defined as a walking speed in the lowest 20% of the distribution, taking account of sex and height; and low physical activity was defined as activity in the lowest sex-specific 20% of the distribution. To avoid the potential problem of spurious shared variance between our measures of frailty status and allostatic load, we omitted the component ‘weight loss’ (defined here as a BMI <18.5 kg) when summing the number of frailty components that were present, on the grounds that BMI is a component of our allostatic load measure.

#### Allostatic load

We used data on nine biomarkers: fibrinogen, triglyceride, ratio of high density to total cholesterol, albumin, glycated haemoglobin, C reactive protein, BMI, and mean systolic blood pressure (SBP) and diastolic BP (DBP). Blood samples (non-fasting) were taken. SBP and DBP were calculated as the average of three sitting readings taken using an Omron 705IT monitor.

We derived an allostatic load score from the nine biomarkers by giving a score of one for each biomarker where participants were in the high-risk quartile of the distribution, and then summing those scores. Participants who reported taking medication for control of hypertension, diabetes or raised cholesterol were treated as if they were in the high-risk quartile of the distribution of the relevant biomarkers on the grounds that use of medication is an indicator of a history of poorer biological regulation.^38^

### Statistical analysis

We used rank order correlations to examine associations between the characteristics of the sample and frailty status and cumulative allostatic load. Multinomial logistic regression was used to examine associations between intelligence or socioeconomic position in childhood and frailty status. Generalised linear models assuming a Poisson distribution were used to examine associations between childhood characteristics and allostatic load. We used Sobel-Goodman and boot-strapping tests to examine the extent to which potential mediating variables carried the influence of intelligence or socioeconomic position in childhood to the outcomes. Analyses are based on 876 participants (80.4% of those who took part in the first follow-up of the cohort) who had complete data.

## Results

Table 1 shows the characteristics of the participants and the rank order correlation between those characteristics, and both frailty status and allostatic load at age 70 years. In total, 7% were frail and 47% were prefrail. There was a significant positive correlation between greater degree of frailty and allostatic load (ρ=0.248) (online supplementary table S1 shows how the distribution of each component of allostatic load varied by frailty status). In general, the correlations between the childhood and adult characteristics of the participants and these two health outcomes were similar. As children, people with a higher degree of frailty or higher allostatic load had a lower IQ and were less likely to have fathers in a professional or managerial occupation, though there were no associations between level of home environmental deprivation and either frailty status or allostatic load. As adults, people with a higher degree of frailty, or higher allostatic load, were less likely to have had a professional or managerial occupation, had lower educational attainment, were more likely to be former or current smokers, and drank less alcohol. Allostatic load was higher in those who were less active. There was no difference in sex distribution by frailty status, but women tended to have a lower allostatic load.

**Table 1:** Characteristics of the study participants and their rank order correlations with frailty status and allostatic load (n=876)

Characteristic	Mean (SD), median (IQR) or number (%)	Correlation with frailty status†	Correlation with allostatic load†
IQ at age 11 years, mean (SD)	100.9 (14.5)	−0.126***	−0.164***
Female, n (%)	443 (50.6)	−0.002	−0.071*
Father in professional/managerial social class, n (%)	229 (26.0)	0.097**	0.100**
Home environmental deprivation score at age 11 years	−0.16 (−0.38 to 0.15)	−0.028	0.009
Professional/managerial social class, n (%)	502 (57.1)	0.142***	0.149***
Has degree, n (%)	132 (15.1)	−0.161***	−0.181***
Allostatic load, median (IQR)	3 (2–5)	0.248***	–
Frailty status, n (%)		–	0.248***
Not frail	404 (46.0)		
Prefrail	410 (46.8)		
Frail	62 (7.06)		
Smoking status		0.079*	0.147***
Never	386 (44.0)		
Ex	384 (43.8)		
Current	106 (12.1)		
Alcohol units per week, n (%)		−0.074*	−0.070*
None	162 (18.5)		
≤21 (men)/≤14 (women)	562 (64.0)		
≥22 (men)/≥15 (women)	152 (17.4)		
Physical activity, mean (SD)	2.97 (1.10)	–	−0.229***

†Rank order correlations with father's or own social class or highest educational qualification were based on the 5-category version of these variables. As low physical activity is a component of frailty status we do not show the correlation between them. ***p<0.001, **p<0.01, *p<0.05.

The relationships between IQ and indicators of socioeconomic circumstances in childhood and frailty status or allostatic load at age 70 did not differ between the sexes (p for interaction terms >0.5). Analyses were therefore carried out in men and women together, adjusting for sex.

Table 2 shows relative risk (RR) ratios for prefrailty and frailty.

**Table 2:** Relative risk ratios† for incident prefrailty or frailty according to intelligence and socioeconomic circumstances in childhood

Model		Relative risk ratios (95% CI)	
		Prefrailty	Frailty
1	Intelligence at age 11, per SD decrease	1.25 (1.08 to 1.45)**	1.57 (1.21 to 2.03)***
2	Father's social class	1.12 (0.97 to 1.30)	1.48 (1.12 to 1.96)**
3	Environmental deprivation at age 11, per SD increase	1.01 (0.78 to 1.31)	0.81 (0.46 to 1.40)
4	Intelligence at age 11, per SD decrease	1.23 (1.06 to 1.44)**	1.48 (1.14 to 1.93)**
	Father's social class	1.07 (0.92 to 1.25)	1.36 (1.02 to 1.82)*
5	Intelligence at age 11, per SD decrease	1.16 (0.98 to 1.37)	1.13 (0.83 to 1.54)
	Father's social class	1.04 (0.89 to 1.21)	1.19 (0.86 to 1.61)
	Highest social class in adulthood	1.14 (0.95 to 1.38)	1.33 (0.91 to 1.54)
	Highest educational qualification	1.00 (0.87 to 1.15)	0.70 (0.52 to 0.96)*
	Smoking status		
	Never	1.0	1.0
	Ex	1.32 (0.97 to 1.79)	1.10 (0.59 to 2.03)
	Current	1.35 (0.84 to 2.15)	1.68 (0.75 to 3.70)
	Units of alcohol per week		
	None	1.0	1.0
	≤21 (men)/≤14 (women)	0.77 (0.52 to to 1.13)	0.48 (0.25 to 0.95)*
	≥22 (men)/≥15 (women)	0.74 (0.44 to 1.17)	0.50 (0.20 to 1.23)

***p<0.001, **p<0.01, *p<0.05.

†All models are adjusted for sex.

Models 1–3 show estimates for childhood cognitive and social risk factors separately, adjusted for sex. Lower intelligence in childhood was associated with an increased risk of being either prefrail or frail. For a SD decrease in IQ, the RRs (95% CIs) were 1.25 (1.08 to 1.45) and 1.57 (1.21 to 2.03), respectively (model 1). Having a father in a lower social class in childhood was associated with an increased risk of frailty: for one category decrease in father's social class, the RR (95% CI) of frailty was 1.48 (1.12 to 1.96) (model 2). People whose father had been in a lower social class also had a slightly increased risk of prefrailty, but this was not significant. There was no association between level of environmental deprivation in the home in childhood and risk of prefrailty or frailty (model 3). When childhood IQ and father's social class were entered in a model together, childhood IQ remained a predictor of prefrailty and frailty, and father's social class remained a predictor of frailty (model 4). We then examined whether the associations between childhood IQ and father's social class on frailty risk were accounted for by attained social class, highest educational qualification or the health behaviours, smoking and alcohol intake (model 5). The inclusion of these factors in the model attenuated the associations between childhood intelligence and prefrailty or frailty, such that both ceased to be significant. The inclusion of these factors also attenuated the association between father's social class and frailty, so it was no longer significant.

Sobel-Goodman tests showed that highest educational qualification, but not attained social class or health behaviours, had a significant independent mediating effect on the association between childhood intelligence and frailty status: 35% of the total effect was mediated through highest educational qualification (p=0.01). Use of a bootstrap test of mediation produced identical results. In total, 28% of the total effect of father's social class on frailty status was mediated through highest educational attainment after controlling for childhood intelligence. Attained social class and health behaviours had no mediating effect.

Table 3 shows the coefficients (95% CIs) for allostatic load according to intelligence and socioeconomic circumstances in childhood.

**Table 3:** Coefficients† (95% CIs) for allostatic load according to intelligence and socioeconomic circumstances in childhood

Model		Coefficient (95% CI)
1	Intelligence at age 11, per SD decrease	0.10 (0.07 to 0.14)***
2	Father's social class	0.06 (0.02 to 0.10)**
3	Environmental deprivation at age 11, per SD increase	0.01 (−0.05 to 0.08)
4	Intelligence at age 11, per SD decrease	0.09 (0.06 to 0.13)***
	Father's social class	0.04 (−0.001 to 0.08)
5	Intelligence at age 11, per SD decrease	0.05 (0.01 to 0.09)*
	Father's social class	0.01 (−0.03 to 0.05)
	Highest social class in adulthood	0.01 (−0.03 to 0.06)
	Highest educational qualification	−0.05 (−0.08 to −0.01)*
	Smoking status	
	Never	Reference
	Ex	0.13 (0.05 to 0.21)**
	Current	0.16 (0.05 to 0.27)***
	Units of alcohol per week	
	None	Reference
	≤21 (men)/≤14 (women)	−0.13 (−0.22 to −0.03)**
	≥22 (men)/≥15 (women)	−0.13 (−0.25 to −0.01)*
	Physical activity	−0.11 (−0.14 to −0.07)***

***p<0.001, **p<0.01, *p<0.05.

†All models are adjusted for sex.

Models 1–3 show estimates for childhood cognitive and social risk factors separately, adjusted for sex. Allostatic load was significantly higher in participants with lower intelligence in childhood. For a SD reduction in IQ, allostatic load increased by 0.10 (0.07 to 0.14). Having a father in a lower social class was associated with increased allostatic load, but there was no association between household deprivation in childhood and allostatic load. When childhood IQ and father's social class were entered in a model together, childhood IQ remained a predictor of allostatic load, but the relation between father's social class and allostatic load was no longer significant (model 4). We then examined whether the associations between childhood IQ and allostatic load were accounted for by attained social class, highest educational attainment or the health behaviours, smoking, alcohol intake and physical activity (model 5). The association between childhood IQ and allostatic load was slightly attenuated by these adjustments but remained significant. Having lower educational attainment, lower physical activity, ever smoking and not drinking alcohol were also independently associated with increased allostatic load. Attained social class was not associated with allostatic load. Sobel-Goodman mediation tests showed that 29% of the total effect of childhood IQ on allostatic load was mediated by educational attainment (p=0.01). Health behaviours and attained social class had no significant mediating effects.

## Discussion

In this study, significant associations between lower intelligence and greater socioeconomic disadvantage in childhood—as indicated by father's social class—and increased risk of frailty at age 70 were attenuated and no longer significant after adjustments for the potential mediating factors, educational attainment, attained social class and health behaviours in adulthood. Of these, only educational attainment had a significant mediating effect. Lower intelligence, but not father's social class, remained a significant predictor of greater allostatic load at age 70 in the fully adjusted model; this was partially mediated through educational attainment.

Chronic stress in early life—such as that entailed by socioeconomic disadvantage—may contribute to accelerated biological ageing.^23^ ^39^ Here, we found that an association between lower childhood social class and increased risk of frailty was mediated through educational attainment. This is consistent with a study where poorer childhood living standards were linked with greater frailty, but not independently of markers of later life socioeconomic position.^5^ Childhood socioeconomic disadvantage may also increase susceptibility to frailty via effects not examined here, such as adult physical activity, obesity and cardiovascular disease.^11^ ^34^ ^40^ We found little evidence in support of our hypothesis that allostatic load would be higher in those exposed to childhood socioeconomic disadvantage. Although allostatic load was slightly higher in participants from lower childhood social classes, this association was confounded by childhood intelligence, and ceased to be significant after adjustment for this factor. The weak association found here between allostatic load and childhood social class is consistent with findings in a study of three cohorts where associations between life course socioeconomic position and allostatic load were only present in the younger two cohorts.^27^ The fact that we found no associations between our other measure of childhood socioeconomic circumstances—environmental deprivation in the home—and either frailty or allostatic load may mean that our measure of environmental deprivation provided a less accurate indicator of socioeconomic disadvantage than father's social class.

Although we found an association between lower childhood intelligence and risk of frailty in a model adjusted for the confounding effect of father's social class, after further adjustment for the potential mediators, the associations between childhood IQ and frailty status were no longer significant. Several studies have shown that frailty occurs more commonly in people of lower socioeconomic status^12^ or educational attainment,^13^ and there is evidence to link it with smoking.^10^ Here we found that educational attainment was the only significant mediator of the association between childhood IQ and frailty status. When interpreting this finding, it is important to bear in mind that childhood intelligence is a predictor of educational outcomes,^15^ that are partly heritable, and that they are significantly correlated genetically.^41^ ^42^ The statistical mediation we find here might be because the ‘influence’ of intelligence is mediated via education, but it is also possible that education is a proxy for intelligence, and that including it in the model is an overadjustment.

Our observation that older people with lower intelligence in childhood have a higher allostatic load independent of childhood socioeconomic circumstances, is consistent with findings that lower early life intelligence was associated with greater physiological dysregulation in midlife.^43^ In this study, there was no exploration of mediating factors. Here we found that the association was partially mediated by educational attainment. Intelligence is thought to play an important part in determining what individuals perceive is stressful,^18^ and is also likely to influence their likelihood of exposure to stressful work or living environment, and the efficacy with which they respond to adversities.^19^ These factors may partly explain the link between childhood IQ and later allostatic load.

The two outcomes in our study, frailty and allostatic load, were significantly correlated with each other (r=0.248). The cross-sectional nature of this association makes it impossible to determine whether higher allostatic load increases risk of frailty—as has been demonstrated previously^25^—or reflects the physiological dysregulation in multiple systems that is characteristic of frailty.^2^ Only one other study has shown an association between higher allostatic load and frailty, and that too was cross-sectional.^44^

Our study has some weaknesses. Information on childhood circumstances was obtained from the participants at age 70 so may be less accurate than parent-derived information in childhood. When deriving the Fried frailty phenotype, we had no data on unintentional weight loss. The original phenotype of frailty studies^1^ used BMI <18.5 kg/m^2^ as a substitute indicator of weight loss, but as BMI was part of our allostatic load measure, we decided against using this as a component of frailty to avoid introducing spurious shared variance between our measures of frailty status and allostatic load. Finally, our measure of allostatic load included non-fasting lipid measurements and contained no biomarkers of neuroendocrine functioning, such as cortisol. Allostatic load measures typically include information for parameters of all major physiological regulatory systems.^45^ The measure used here might more accurately be viewed as a measure of cardiometabolic risk.

In this prospective study, significant associations between lower intelligence and greater socioeconomic disadvantage in childhood—as indexed by father's social class—and increased risk of frailty at age 70 years were attenuated and ceased to be significant when adjusted for potential mediating factors. Both associations were mediated through educational attainment. Lower intelligence, but not social class in childhood, was associated with higher allostatic load, a correlate of frailty status, and this association, though attenuated, remained significant in the fully adjusted model. Part of this association was mediated through educational attainment. Further research will need to investigate the mechanisms whereby lower childhood intelligence is linked to greater physiological dysregulation in later life.

**What is already known on this subject**

- Frailty is a syndrome observed in older people whose core feature is increased vulnerability to stressors due to dysregulation in multiple physiological systems.
- Allostatic load is a measure of multisystem physiological dysregulation that reflects the effects of exposure to chronic stress.
- Evidence on childhood determinants of frailty or allostatic load in later life frailty is sparse, but there are indications that greater socioeconomic disadvantage in childhood may increase the risk of frailty and lead to higher allostatic load.

**What this study adds**

- Associations between lower intelligence and greater socioeconomic disadvantage in childhood—as indexed by father's social class—and increased risk of frailty at age 70 years were attenuated and no longer significant after adjustment for potential mediating factors, in particular educational attainment.
- Lower intelligence, but not socioeconomic disadvantage, in childhood was also associated with higher allostatic load at age 70 years, and this persisted, though attenuated, after adjustment for potential mediating factors. Educational attainment partially mediated this association.

## Supplementary Material

Web supplement
