# Supplementary material for: Intelligence and socioeconomic position in childhood in relation to frailty and cumulative allostatic load in later life: the Lothian Birth Cohort 1936
Source: J Epidemiol Community Health. 2015 Dec 23;70(6):576–82. doi: 10.1136/jech-2015-205789 (PMC4820036; doi:10.1136/jech-2015-205789)
Supplement: Web supplement [file jech-2015-205789-s1.pdf]

Supplementary table

Percentage of study participants in the high-risk quartile of each component of the allostatic load measure according to frailty status

High-risk quartile	Not frail (n=404)	Pre-frail (n=410)	Frail (n=62)	P value
Systolic blood pressure	56.7	56.8	77.4	0.006
Diastolic blood pressure	56.9	57.5	79.0	0.003
HbA1c	18.6	30.6	51.6	<0.001
Triglycerides	32.7	46.1	64.5	<0.001
HDL to total cholesterol ratio	22.8	23.1	17.1	0.640
BMI	14.6	30.3	46.7	<0.001
Fibrinogen	21.8	34.2	43.6	<0.001
Albumin	31.7	36.4	43.6	0.117
